# Rewriting the crime divide: how executive functions and social cognition processes challenge violence classifications in the Colombian prison population

**DOI:** 10.3389/fpsyg.2026.1741171

**Published:** 2026-04-01

**Authors:** Maria Teresa Cuervo Cuesta, Maria Antonia Chagüendo-Quintero, Natalia Cadavid-Ruiz, Carlos Alberto Dorado-Ramírez, Eduar Herrera Murcia, Mateo Belalcázar Correa, Merce Jodar Vicente

**Affiliations:** 1 Department of Social Sciences, Pontificia Universidad Javeriana, Cali, Colombia; 2 Department of Clinical and Health Psychology, Autonomous University of Barcelona, Barcelona, Spain; 3 Centro de Estudios Cerebrales Universidad del Valle, Cali, Colombia; 4 Departamento de Estudios Psicológicos, Universidad Icesi, Cali, Colombia; 5 Instituto de Investigación en Ciencias del Desarrollo, del Aprendizaje y Subjetividades (CIDEAS), Universidad del Valle, Cali, Colombia; 6 Department of Clinical and Health Psychology, Universitat Autònoma de Barcelona, Barcelona, Spain; 7 Department of Neurology, Institut d’Investigació i Innovació Parc Taulí, Hospital Universitari Parc Taulí, Sabadell, Spain; 8 Centro de Investigación Biomédica en Red–Salud Mental (CIBERSAM), Instituto Carlos III, Madrid, Spain

**Keywords:** executive functions, inmates, prison, social cognition, violent behavior

## Abstract

**Background:**

Executive functions (EF) and social cognition (SC) are fundamental to self-regulation and social behavior. Their impairment has been associated with criminal behavior, yet few studies integrate both areas, especially in highly violent contexts such as Colombia.

**Objective:**

This observational study compared neuropsychological performance in EF (flexibility, working memory, and inhibition) and SC (emotional recognition and empathy) among males convicted of violent crimes (VC), nonviolent crimes (NVC), and a control group (CG).

**Methods:**

A total of 117 men (39 per group), matched for age and educational level, participated in a case-control design. Standardized tests were administered: M-WCST, WAIS-IV subscales, Stroop, Mini-SEA, and Pain Empathy Task.

**Results:**

Both groups of inmates performed worse on EF compared to the CG, with the difference being significant only in working memory. On SC, the NVC group showed better recognition of negative emotions, while the VC group was able to identify the emotion of sadness more accurately. Both groups showed greater affective empathy and empathic concern than the CG, and the VC group stood out for its cognitive empathy.

**Conclusion:**

These findings show diverse profiles that don’t fit into the violent/nonviolent classification, highlighting the complexity of criminal behavior and the need to expand research with integrative approaches that allow for a better understanding of criminal behavior from the perspective of cognitive neuroscience.

## Introduction

Violence represents a global public health crisis, claiming more than 1.3 million lives annually, which constitutes an alarming 2.5% of global mortality (Organización Mundial de la Salud [OMS], 2014). Countries such as Ecuador (44.5 per 100,000 inhabitants), Honduras (31.1), Venezuela (26.8), Mexico (23.3), and Colombia (25.7) consistently rank among the most violent according to recent data (Chevalier, 2024; International Rescue Committee, 2022), highlighting an extensive and deep-rooted problem.

In Colombia, such violence translates into alarming crime statistics. In 2024, there were 13,393 homicides reported, in addition to thousands of robberies and other violent crimes (Instituto Nacional Penitenciario y Carcelario [INPEC], 2025; Insight Crime, 2025), reflecting an everyday reality of insecurity and transgression. A direct consequence is the exponential growth of the prison population, which in Colombia will exceed 104,000 people in 2026, placing it among the highest in the region alongside with Brazil (Instituto Nacional Penitenciario y Carcelario [INPEC], 2025; World Prison Brief, 2025).

This overpopulation generates a critical overcrowding rate (34%), overwhelming the capacity of the prison system and severely limiting the possibilities for rehabilitation (Baracaldo, 2013; Instituto Nacional Penitenciario y Carcelario [INPEC], 2025). This scenario, coupled with high recidivism rates, imposes an unsustainable social and economic burden on public safety and perpetuates cycles of violence and exclusion (Insight Crime, 2020). The prison environment itself, often impoverished and stressful, can exacerbate physical and mental health problems, and even reduce cognitive functions key to self-regulation, contributing to recidivism (Causadias et al., 2010; Cruz et al., 2020; Meijers et al., 2017, 2023).

From a neuropsychological perspective, criminal behavior is thought to result from the interaction between individual brain function and social environment conditions (Altimus, 2016; Bigenwald and Chambon, 2019). This approach allows the identification of specific neurocognitive processes that may contribute to the risk of social transgressions. These include executive functions (EF), mediated mainly by the prefrontal cortex, which comprise processes such as cognitive flexibility, working memory, and inhibitory control (Tirapu-Ustárroz et al., 2017).

Similarly, social cognition (SC), which includes emotional processing and empathy, plays an essential role in the self-regulation of interpersonal and moral behavior (Baez et al., 2017; Bennet et al., 2005). Both domains are fundamental for emotional self-regulation, inhibition of impulsive responses, and adaptation to social norms. In fact, it has been proposed that the development of SC depends, in part, on the maturation of executive control, given that both share functional substrates in the prefrontal cortex (Adolphs, 2003; Blakemore et al., 2007; Smolker et al., 2022). In this regard, it has been suggested that the progressive strengthening of executive control could facilitate the parallel development of complex social skills such as perspective taking and empathic regulation (Blakemore et al., 2007; Moriguchi et al., 2007).

In line with the above, previous research has found lower EF performance among individuals who have committed a crime compared to the average population, particularly in the planning and monitoring of tasks (Delfín et al., 2018; Greenfield and Valliant, 2007; Hanlon et al., 2010; Meijers et al., 2017; Moreno, 2014; Ross and Hoaken, 2011; Santos-Barbosa and Coelho-Monteiro, 2008; Seruca and Silva, 2015); in cognitive flexibility (Arana et al., 2013; Hanlon et al., 2010; Herrero et al., 2010; Karlsson et al., 2016; Meijers et al., 2015; Seruca and Silva, 2015; Wallinius et al., 2019); in working memory (Moreno, 2014); and inhibition (Causadias et al., 2010; Meijers et al., 2017, 2018; Wallinius et al., 2019).

In the study of cognitive impairment in prisoners, alterations have been described in several brain structures including the hippocampus, insula, amygdala, orbitofrontal cortex, somatosensory cortex, and frontal lobe, all of which play a significant role in criminal behavior (Bertone et al., 2017). Areas such as the frontal lobe, which is essential for impulse control, exhibit alterations in their structure varying from disconnection or blockage to overexcitement and hyperactivity, leading to poor self-regulation that can result in unusual behavior (Bertone et al., 2017). In addition, severe and moderate anatomical and functional alterations have been observed in the dorsolateral cortex (Jiménez and Robledo, 2011).

Executive dysfunction has been linked to violent crimes, where poor planning skills, mental inflexibility or rigidity, low verbal intelligence, and attention deficits reduce the ability to cope with both internal and external stressors, which can lead to aggressive or violent behavior due to poor regulation of negative affect and inhibitory control (Fontao and Ross, 2021). Similarly, working memory deficits may compromise the ability to hold in mind the potential consequences of one’s actions, to update behavioral plans in response to changing social cues, and to regulate emotional responses during conflict situations, thereby increasing the likelihood of impulsive or aggressive reactions (Meijers et al., 2017; Rajtar-Zembaty et al., 2017). In this sense, working memory acts not only as a cognitive resource for information processing, but also as a mediator between emotional regulation and behavioral control in social contexts. Furthermore, it has been shown that the dysfunctional brain network in violent offenders critically involves the cerebellum and underlies moral behavior and violence (Leutgeb et al., 2016).

In turn, difficulties have been reported in the SC of individuals with criminal records (Stams et al., 2006; Quintero et al., 2021), such as altered social perceptions and socially inappropriate responses (Bertone et al., 2017; Fortuny et al., 2023; Labbé et al., 2019), in addition to alterations in theory of mind (Brook and Kosson, 2013; Drayton et al., 2018; Girolamo et al., 2022; McBride and Ridinger, 2016), moral judgment/moral reasoning (Butman, 2001; Colby and Kohlberg, 1987; Greenfield and Valliant, 2007), emotional processing or facial emotion recognition (Bertone et al., 2017; Pinedo and Yáñez, 2017; Pino et al., 2019; Philipp-Wiegmann et al., 2017) and empathy (Brook and Kosson, 2013; Mayer et al., 2018; Quintero et al., 2021).

Similarly, structures such as the occipitotemporal cortex, amygdala, orbitofrontal cortex, basal ganglia, and right parietal cortex are involved in facial emotion recognition (Adolphs, 2002), with the left parahippocampal area being particularly prominent in men (Proverbio, 2021). Neuroimaging in adults confirms that the areas of the brain activated when viewing facial expressions of pain in others are the same as those activated when experiencing pain oneself, including the anterior cingulate cortex, the anterior insula, supplementary motor area, amygdala, somatosensory cortex, and periaqueductal gray area (Legrain et al., 2011).

Structures and systems such as the brainstem, amygdala, hypothalamus, striatum, insula, anterior cingulate cortex, and orbitofrontal cortex are involved in empathy (Quintero et al., 2021). In cognitive empathy, there are structures linked to perspective-taking, with greater activation in the anterior left dorsomedial prefrontal gyrus and the left supramarginal gyrus. In affective empathy, there are areas associated with the subjective response of feelings and the imitation of emotions, with the posterior left dorsomedial prefrontal gyrus and the left inferior frontal gyrus showing the greatest excitation (Kogler et al., 2020).

Despite growing interest in understanding the neuropsychological factors associated with criminal behavior, few studies have jointly assessed EF and SC, differentiating between types of crimes such as violent (V) and nonviolent (NV) crimes (Allain et al., 2020; Seidl et al., 2020; Yan et al., 2019). This omission is particularly relevant in countries such as Colombia, where the prison population is growing steadily and faces high levels of structural violence (Instituto Nacional Penitenciario y Carcelario [INPEC], 2025; World Prison Brief, 2025). Although there is evidence of the impact of chronic exposure to violent and criminal contexts on cognitive functioning, inmates remain a poorly studied population, despite their high vulnerability and social marginalization (Chaguendo-Quintero et al., 2023; Meijers et al., 2017, 2018).

From a neuropsychological perspective, examining EF and SC within incarcerated populations contributes to a broader global effort to conceptualize crime through a public-health and developmental lens, emphasizing how cognitive vulnerabilities interact with chronic exposure to social adversity and structural violence. This can provide a better understanding of the mechanisms underlying criminal behavior, a contribution far beyond the Colombian context (Chaguendo-Quintero et al., 2023; Clark et al., 2020). Moreover, it may be key to transforming prisons into spaces for strengthening adaptive social skills, rather than perpetuating punitive environments that encourage violent behavior and deteriorate the mental health of incarcerated individuals (Chaguendo-Quintero et al., 2023; Clark et al., 2020).

The present study hypothesized that individuals incarcerated for violent crimes would exhibit greater impairments in EF and SC compared to the non-violent crime group and the control group, while the latter would show performance within normal parameters or significantly higher than that of the violent crime group. Within this framework, the present study aimed to describe the neuropsychological profile of EF (cognitive flexibility, inhibition, and working memory) and SC (emotional recognition and empathy) among incarcerated men in Colombia convicted of violent and nonviolent crimes. As well as, to compare their performance with a control group, in order to identify possible functional differences associated with the type of crime.

## Materials and methods

### Design

This study is an analytical observational investigation with a matched case-control design and a cross-sectional approach. This type of design allows for the comparison of the behavior of dependent variables between groups previously defined according to the exposure or condition of interest, in this case, the type of crime committed compared to controls (Manterola et al., 2019). Therefore, the differences between the groups were analyzed at a single point in time, without intervening in the exposure or modifying the characteristics of the participants.

### Participants

The sample size was calculated using the G-Power program (version 3.1) for the one-way ANOVA statistical test with three groups. A statistical power of 0.95, an alpha of 0.05, and an expected effect size of 0.4 were considered. The final sample was composed by 117 men, divided into three groups: (a) 39 subjects convicted of aggravated homicide or rape, referred to as violent crimes (V); (b) 39 subjects convicted of petty theft and drug possession, referred to as non-violent crimes (NV); and (c) 39 men with no criminal record or history of incarceration, referred to as the control group (CG).

The inclusion criteria for the VC and NVC groups were: (a) being male; (b) ages between 18 and 45 years old; (c) having been convicted of a crime (violent or nonviolent); (d) having completed at least fifth grade of elementary school; and (e) having served at least 1 year in prison. For the CG, the inclusion criteria were: (a) being male; (b) ages between 18 and 45 years old; (c) never been incarcerated; (d) no criminal record; and (e) having completed at least fifth grade of elementary school.

The exclusion criteria applicable to all groups were: (a) having an IQ below 75 points, the Spanish version of the National Adult Reading Test (TAP); (b) having a score corresponding to the severe range (≥26 points) on the Beck Anxiety Inventory (BAI) and the Beck Depression Inventory-II (BDI-II); (c) having a history of neurological and/or psychiatric disorders reported in the medical record; (d) having a current diagnosis of psychoactive substance use disorder; and (e) being under pharmacological treatment with effects on the central nervous system. For the VC and NVC groups, those who had committed multiple different crimes were also excluded.

A total of 183 individuals were evaluated. Of these, 66 were excluded during the screening phase for not meeting the inclusion criteria, for presenting exclusion criteria, or for not completing the evaluation. In order to ensure the control and internal validity of the study, the groups were matched, resulting in a final sample of 117 participants, distributed in groups of 39. One-to-one matching with the VC and NVC groups was performed based on age, level of education, and IQ.

The CG was intentionally recruited from two higher education institutions, specifically from among general services staff who met the necessary characteristics for matching in terms of age, educational level, and IQ. The selection was made using non-probability convenience sampling, seeking to maintain a one-to-one correspondence with the incarcerated participants. To ensure the absence of criminal records, a double verification was carried out: (a) direct verbal self-report by the participants, and (b) consultation of the freely accessible public platform for the verification of criminal records. Likewise, a brief screening interview was used to explicitly inquire about personal history of psychiatric illness, complementing this information with the application of screening instruments for symptoms of anxiety and depression. These strategies strengthened the control of confounding variables and the internal validity of the study.

Table 1 presents the sociodemographic and screening data of the participants. No significant differences were found between groups with respect to age [*F*(2, 114) = 3.04, *p* = 0.052] and years of education [χ^2^ (2) = 10.87, *p* = 0.093]. Similarly, there were no differences between the VC and NVC groups in the number of convictions [χ^2^ (3) = 4.27, *p* = 0.234], although significant differences were found in the length of imprisonment in months depending on the type of crime committed [*F*(1, 76) = 21.51, *p* < 0.001]. Likewise, no significant differences were found in anxiety scores [*H*(2) = 0.80, *p* = 0.669], depression [*H*(2) = 2.67, *p* = 0.263], and IQ [*F*(2, 114) = 1.36, *p* = 0.259].

**TABLE 1:** Sociodemographic and screening variables.

Variables	VC		NVC		CG	
	*M*	*SD*	*M*	*SD*	*M*	*SD*
Gender, male–n (%)	39 (100)		39 (100)		39 (100)	
Age	32.95	5.7	30.23	6.45	29.05	8.92
Years of education	10.94	1.19	10.76	1.45	11.69	1.84
Number of times in prison	1.18	0.51	1.46	0.85	-	-
Time spent in prison in months ***	69.23	31.01	38.01	28.37	-	-
Anxiety (BAI)	3.08	3.77	2.95	4.96	3.77	4.84
Depression (BDI)	4.64	6.17	4.1	4.42	5.85	5.37
Intelligence quotient (IQ-TAP)	17.77	4.18	15.72	3.87	17.21	4.2

***p < 0.001. M, Mean; SD, Standard Deviation; VC, Violent Crimes; NVC, Non-Violent Crimes; CG, Control Group.

### Instruments

Three instruments were used as screening tests to assess the presence and frequency of anxiety and depression symptoms, as well as to estimate the participants’ IQ. To evaluate affective symptoms, the Beck Anxiety Inventory (BAI) and the Beck Depression Inventory (BDI) were used, both consisting of 21 items and with high internal consistency indices (α ≥ 0.85) (Sanz, 2011; Sanz and Vázquez, 2011). To estimate IQ, the Spanish version of the National Adult Reading Test (TAP) was used, which has proven to be a reliable method (α = 0.84) for assessing IQ in Spanish-speaking populations (Pluck et al., 2017).

The EFs were assessed using three tests. Cognitive flexibility was measured using the modified version of the Wisconsin Card Sorting Test (M-WCST), with a reported reliability between α = 0.70 and 0.80 (Ojeda et al., 2019). In this test, a higher number of total errors and perseverative responses is an indicator of impaired cognitive flexibility, while a higher number of completed categories reflects better performance. Working memory (WM) was estimated using the Digits (D) and Number-Letter (NL) subtests of the Wechsler Adult Intelligence Scale (WAIS-IV), with reliability coefficients ranging from α = 0.88 and 0.93 (Wechsler, 2012). Inhibitory control and interference resistance were assessed using the Stroop Color and Word Test, an instrument with a reliability of α = 0.88 (Golden, 2020).

The SC was examined using two instruments. Recognition of basic facial emotions was assessed using the corresponding subtest of the Mini-SEA, based on Ekman’s faces, with high reliability (α = 0.87; ICC = 0.85–0.92) (Bertoux et al., 2012, 2014). Empathy was measured using the computerized Empathy for Pain Task, which presents 24 scenarios involving intentional, accidental, and neutral situations and has shown internal consistency of α = 0.73 and inter-rater reliability greater than α = 0.80 (Baez et al., 2015). This test assesses empathy through eight components, three of which were considered in this study: affective empathy, cognitive empathy, and empathic concern. This decision was consistent with the objectives of the research, which focused specifically on assessing social cognition in terms of empathic processing, rather than moral judgments or punitive attributions. This avoided the inclusion of conceptually distinct variables (e.g., punishment or moral judgment), allowing for greater conceptual precision, a reduction in the number of statistical comparisons, and a more parsimonious analysis aligned with the theoretical framework.

For interpretation purposes, it should be noted that higher scores on the M-WCST in correct categories indicate better performance, whereas higher scores in perseverations and errors reflect greater cognitive rigidity. On the WAIS-IV subtests, higher scores indicate better working memory performance. For the Stroop test, higher scores on Word, Color, and Word-Color trials indicate faster processing, while a higher Interference score reflects greater inhibitory control capacity.

### Procedure

Once the research project was approved by the National Penitentiary and Prison Scientific Research Group (INPEC) and by the Research and Ethics Committee of the executing institution, potential participants were preselected by identifying, within the prison population, individuals convicted of VC and NVC who met the inclusion and exclusion criteria. From this initial list, 78 participants (39 per group) were randomly selected and invited to participate in the research.

During the first meeting, the objective of the research and the evaluation procedure were explained to them. Those who agreed to participate signed the informed consent form and continued with the screening tests. Those who met the established criteria advanced to the neuropsychological testing phase. These tests were administered individually, in a single session lasting approximately 1 h. The CG was intentionally selected from the general population and matched with the two groups of prisoners in terms of age, educational level, and IQ. This group was evaluated under similar conditions.

### Data analysis

Data analysis was performed using the statistical program RStudio. Descriptive and inferential statistics were calculated to analyze performance on the EF and SC tests. To facilitate visualization and comparison between groups, the normative scores obtained on each test were transformed into Z scores and represented using waterfall charts. The analyses considered the comparison between the three groups in relation to the domains of EF (cognitive flexibility, working memory, and inhibitory control) and SC (facial emotion recognition and empathy).

Data distribution was assessed using Kolmogorov-Smirnov and multivariate normality tests (Mardia and Henze-Zirkler). Based on these results, parametric analyses (MANOVA/ANOVA with Tukey’s HSD; η^2^*p*) were applied to normally distributed executive measures (WAIS-IV Digits and Stroop). Conversely, non-parametric Kruskal-Wallis tests (followed by Wilcoxon pairwise comparisons; ε^2^) were employed for variables violating normality assumptions, including WCST, WAIS-IV Number-Letters, and all social cognition tasks (Mini-SEA and Pain Task). *Post-hoc* analyses included the Tukey test (parametric analyses) and the Wilcoxon test (nonparametric analyses). All pairwise *post-hoc* comparisons were adjusted using the Holm sequential correction to control the family-wise error rate arising from multiple testing (Holm, 1979).

To assess whether the length of incarceration acted as a confounding variable, three complementary analyses were conducted: (1) Spearman correlations between incarceration length and all cognitive variables across the prisoners; (2) ANCOVAs comparing the VC and NVC groups, controlling for incarceration length as a covariate; and (3) three-group ANCOVAs (VC + NVC + Control) assigning a value of 0 to the control group to simulate a design unconfounded by time. These analyses systematically evaluated whether the observed group differences could be explained by differential incarceration lengths rather than by premorbid neurocognitive profiles associated with the type of offense.

### Ethical considerations

This project was developed in accordance with the ethical principles established in the Declaration of Helsinki (Declaración de Helsinki de la AMM, 2013) and current Colombian regulations for research involving human subjects, including Resolutions 8,430 of 1993 and 2,,378 of 2008. The guidelines of Law 1,090 of 2006 on the professional practice of psychology, international agreements on ethics in human research, and the intellectual property guidelines of the Vancouver Group were also followed. In accordance with national regulations (Article 11, paragraph b, Resolution 008430 of the Ministry of Health), this research was classified as posing minimal risk to participants.

## Results

**FIGURE 1:**
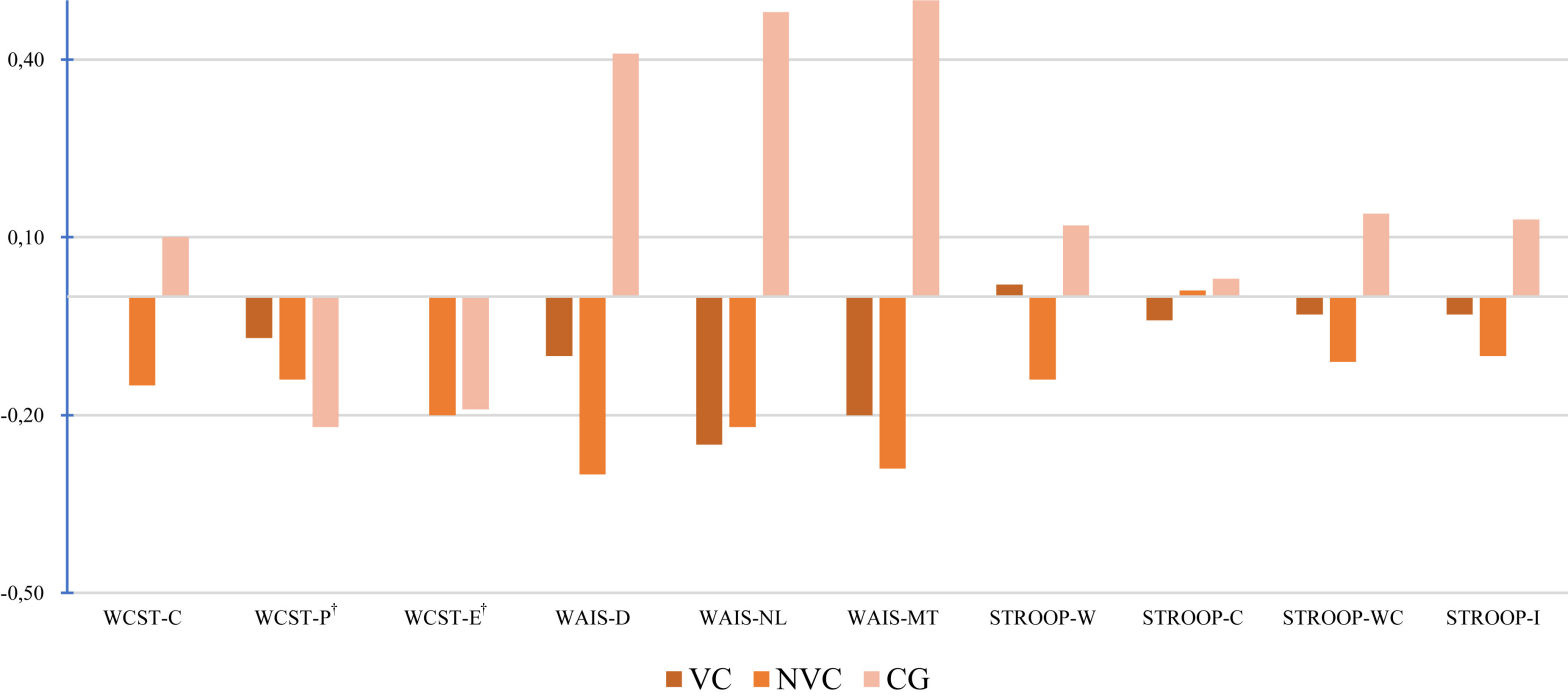
Z-score profile of EFs across study groups. VC, Violent Crimes; NVC, Non-Violent Crimes; CG, Control Group; WCST-C, Correct categories; WCST-P, Perseverations†; WCST-E, Errors†; WAIS IV-D, Digits; WAIS IV-NL, Numbers and Letters; WAIS IV-WM, Working Memory Sum; STROOP-W, Word; STROOP-C, Color; STROOP-WC, Word-Color; STROOP-I, Interference. Z-scores were computed from normative data to enable comparison across tests with different metrics. † For Perseverations and Errors, higher scores indicate worse performance.

The profile of the EFs showed different patterns between groups. In the cognitive flexibility task (M-WCST), the NVC group performed below average, as reflected in greater perseveration and errors, as well as a lower number of correctly classified categories. In working memory (Direct and Indirect Digits/Letters and Numbers from the WAIS-IV), both groups of prisoners scored lower than the CG. With regard to the inhibition task (Stroop), both groups performed worse, with the NVC group performing worse (Figure 1).

**FIGURE 2:**
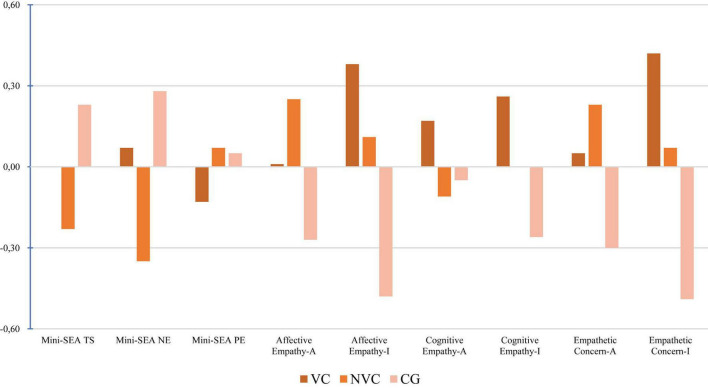
Z-score profile of SC across study groups. VC, Violent Crimes; NVC, Non-Violent Crimes; CG, Control Group; Mini-SEA TS, Total score; Mini-SEA NE, Negative emotions; Mini-SEA PE, Positive emotions; N, Neutral; A, Accidental; I, Intentional. Z-scores were computed from normative data to enable comparison across tests with different metrics.

With regard to SC, in the facial emotion recognition test (Mini-SEA), the NVC group showed better performance in recognizing faces in general and in identifying negative emotions, and similar performance in recognizing positive emotions compared to the CG. In the VC group, the score was above the control group average for recognizing faces in general and negative emotions, but slightly below for positive emotions (Figure 2).

In the Empathy for Pain Task, differential patterns were observed depending on the type of empathy and situation. In affective empathy, the VC and NVC groups outperformed the CG in intentional situations, indicating better affective resonance. The NVC group scored higher in accidental situations. In cognitive empathy, the VC group obtained higher scores in accidental and intentional situations, showing a high capacity to put themselves in the other person’s mind. Empathetic concern was lower in the VC group in neutral situations, but higher in accidental and intentional situations, demonstrating greater recognition of the urgency of caring for the other person’s wellbeing (Figure 2).

Table 2 presents the comparison between the groups in the EF tasks. No significant differences were found in cognitive flexibility (*p* > 0.05). However, significant differences were observed in working memory across all indices (*p* ≤ 0.004). Specifically, the Digits test revealed a medium effect size [η^2^ = 0.09, 95% CI (0.02, 1.00)], while the Number-Letter sequencing and the Total Working Memory Sum both showed substantial effect sizes [ε^2^ = 0.14, 95% CI (0.06, 1.00)]. The Control Group (CG) outperformed both offender groups across all working memory measures. For the Digits subtest, the CG scored higher than the Violent Crimes (VC) group by Δ = +1.72 (direct) and Δ = +1.00 (indirect), and higher than the Non-Violent Crimes (NVC) group by Δ = +1.31 (direct) and Δ = +1.33 (indirect). For Number-Letter sequencing, pairwise effect sizes indicated moderate differences: CG vs. VC, *r* = 0.43 [0.23, 0.62], and CG vs. NVC, *r* = 0.35 [0.14, 0.54]. Similarly, for the Total Working Memory Sum, effect sizes were CG vs. VC, *r* = 0.39 [0.19, 0.58], and CG vs. NVC, *r* = 0.39 [0.18, 0.56]. Pairwise comparisons confirmed that the CG demonstrated superior performance compared to both offender groups, with effect sizes ranging from moderate (*r* = 0.35) to moderate-high (*r* = 0.43). Finally, no significant differences were observed in inhibitory control between groups.

**TABLE 2:** Comparison between groups in EF tests.

Executive functions	Subvariables	VC		NVC		CG		Stat (df)	*p*	ES [95% CI]
		*M*	*SD*	*M*	*SD*	*M*	*SD*			
Flexibility (M-WCST)	Correct categories	5.08	1.29	4.79	1.51	5.15	1.23	H(2) = 1.28	0.529	ε^2^ = 0.01 [0.00, 1.00]
	Perseverations †	4.85	4.25	5.15	5.09	3.56	3.14	H(2) = 1.59	0.452	ε^2^ = 0.01 [0.00, 1.00]
	Errors †	10.15	7.9	11.69	8.4	8.64	6.26	H(2) = 2.32	0.313	ε^2^ = 0.02 [0.00, 1.00]
Working memory (WM-WAIS-IV)	Digits	24.38	3.05	23.39	3.35	26.1	3.21	*F*(2, 114) = 5.85	0.004**	η^2^ = 0.09 [0.02, 1.00]
	Number-letters	15.56	3.35	15.69	4.14	18.18	2.32	H(2) = 16.33	< 0.001***	ε^2^ = 0.14 [0.06, 1.00]
	WM sum	39.95	5.8	39.38	6.26	44.28	5.01	H(2) = 15.85	< 0.001***	ε^2^ = 0.14 [0.06, 1.00]
Inhibitory control (STROOP)	Word (W)	98.23	16.01	95.54	16.34	99.77	14.51	*F*(2, 114) = 0.73	0.483	η^2^ = 0.01 [0.00, 1.00]
	Color (C)	66.03	9.55	66.74	10.41	66.95	12.95	*F*(2, 114) = 0.08	0.928	η^2^ < 0.01 [0.00, 1.00]
	Word-color (WC)	39	10.26	38.26	8.97	40.72	9.41	*F*(2, 114) = 0.68	0.509	η^2^ = 0.01 [0.00, 1.00]
	Interference (I)	0.94	6.93	–0.81	7.3	9.31	2.45	*F*(2, 114) = 0.59	0.558	η^2^ = 0.01 [0.00, 1.00]

**p < 0.01,

***p < 0.001. M, Mean; SD, Standard Deviation; VC, Violent Crimes; NVC, Non-Violent Crimes; CG, Control Group; H, Kruskal-Wallis statistic; F, ANOVA F-statistic; ε^2^, rank epsilon-squared; η^2^, eta-squared. Holm-corrected *post-hoc* comparisons were conducted for significant omnibus tests. †Higher scores indicate worse performance.

Table 3 reports differences in facial emotions recognition. Significant differences were found for disgust [ε^2^ = 0.05, 95% CI (0.01, 1.00)], total score [ε^2^ = 0.06, 95% CI (0.02, 1.00)], and negative emotions [ε^2^ = 0.07, 95% CI (0.02, 1.00)]. In all three cases, *post-hoc* analyses indicated that the Non-Violent Crimes (NVC) group performed significantly better than the Control Group (CG), with moderate effect sizes (*r* = 0.28, *r* = 0.29, and *r* = 0.30, respectively; all p adj < 0.05). Additionally, a specific significant difference was observed for sadness [ε^2^ = 0.05, 95% CI (0.02, 1.00)]. In this case, the Violent Crimes (VC) group recognized sadness significantly better than the NVC group [*r* = 0.28, 95% CI (0.08, 0.49), p adj = 0.036]. This result suggests a specific sensitivity to sadness cues in the VC group compared to non-violent offenders.

**TABLE 3:** Comparison between groups in facial emotions recognition.

Facial emotions (mini-SEA)	VC		NVC		CG		Stat (df)	*p*	ES [95% CI]
	*M*	*SD*	*M*	*SD*	*M*	*SD*			
Fear	1.82	1.35	2.26	1.23	1.69	1.38	H(2) = 3.74	0.154	ε^2^ = 0.03 [0.00, 1.00]
Disgust	2.9	1.33	3.38	1.23	2.74	1.19	H(2) = 6.12	0.047*	ε^2^ = 0.05 [0.01, 1.00]
Anger	3	1.08	3.13	1	2.74	1.23	H(2) = 2.70	0.259	ε^2^ = 0.02 [0.00, 1.00]
Surprise	4.05	1.28	4.41	0.85	4.38	1.14	H(2) = 2.32	0.313	ε^2^ = 0.02 [0.00, 1.00]
Sadness	3.41	1.31	2.97	1.39	2.67	1.26	H(2) = 6.25	0.044*	ε^2^ = 0.05 [0.02, 1.00]
Neutral	4.49	1.07	4.51	0.91	4.54	0.79	H(2) = 0.42	0.811	ε^2^ < 0.01 [0.00, 1.00]
Joy	4.92	0.27	4.79	0.41	4.85	0.43	H(2) = 2.66	0.264	ε^2^ = 0.02 [0.00, 1.00]
Total score	24.59	3.96	25.46	3.93	23.62	3.75	H(2) = 7.39	0.025*	ε^2^ = 0.06 [0.02, 1.00]
Negative emotions	11.13	2.73	11.74	2.75	9.85	3.13	H(2) = 7.70	0.021*	ε^2^ = 0.07 [0.02, 1.00]
Positive emotions	8.97	1.35	9.21	0.98	9.23	1.39	H(2) = 1.93	0.381	ε^2^ = 0.02 [0.00, 1.00]

*p < 0.05. M, Mean; SD, Standard Deviation; VC, Violent Crimes; NVC, Non-Violent Crimes; CG, Control Group; H, Kruskal-Wallis statistic; ε^2^, rank epsilon-squared. Holm-corrected *post-hoc* comparisons were conducted for significant omnibus tests.

Table 4 summarizes the results for empathy. Significant differences were found in affective empathy during intentional situations [ε^2^ = 0.16, 95% CI (0.08, 1.00)]; *post-hoc* analyses indicated that the Violent Crimes (VC) group showed greater affective resonance than the Control Group (CG) (*r* = 0.48, *p* < 0.001). Regarding cognitive empathy, differences were significant only in neutral situations (*p* < 0.001). Finally, for empathic concern, the strongest effect was observed in intentional situations [ε^2^ = 0.17, 95% CI (0.09, 1.00)], with the VC group demonstrating significantly higher concern than the CG (*r* = 0.51, *p* < 0.001). Overall, the prisoner groups showed greater empathic sensitivity than the CG, particularly in intentional emotional contexts.

**TABLE 4:** Comparison between groups in empathy components.

Components	Situations	VC		NVC		CG		Stat (df)	*p*	ES [95% CI]
		*M*	*SD*	*M*	*SD*	*M*	*SD*			
Affective empathy	Neutral	–6.23	2.87	–4.27	2.87	–5.10	3.63	H(2) = 3.36	0.186	ε^2^ = 0.03 [0.01, 1.00]
	Intentional	7.59	1.78	6.70	1.78	4.73	3.65	H(2) = 18.44	< 0.001***	ε^2^ = 0.16 [0.09, 1.00]
	Accidental	2.73	4.09	3.80	4.09	1.41	4.68	H(2) = 5.15	0.076	ε^2^ = 0.05 [0.01, 1.00]
Cognitive empathy	Neutral	32.29	16.01	33.82	16.01	43.01	8.19	H(2) = 12.81	0.002**	ε^2^ = 0.11 [0.04, 1.00]
	Intentional	51.28	3.09	49.62	3.09	47.97	7.89	H(2) = 4.55	0.103	ε^2^ = 0.04 [0.01, 1.00]
	Accidental	33.99	8.14	30.51	8.14	31.22	13.85	H(2) = 0.39	0.822	ε^2^ < 0.01 [0.00, 1.00]
Empathetic concern (motivational)	Neutral	–6.26	2.87	–4.45	2.87	–5.00	3.73	H(2) = 3.19	0.203	ε^2^ = 0.03 [0.00, 1.00]
	Intentional	7.73	2.11	6.46	2.11	4.42	4.00	H(2) = 19.93	< 0.001***	ε^2^ = 0.17 [0.09, 1.00]
	Accidental	3.51	4.24	4.36	4.24	1.84	4.62	H(2) = 3.36	0.186	ε^2^ = 0.03 [0.01, 1.00]

**p < 0.01,

***p < 0.001. M, Mean; SD, Standard Deviation; VC, Violent Crimes; NVC, Non-Violent Crimes; CG, Control Group; H, Kruskal-Wallis statistic; ε^2^, rank epsilon-squared. Holm-corrected *post-hoc* comparisons were conducted for significant omnibus tests.

When incarceration length was statistically controlled for using ANCOVAs comparing the VC and NVC groups, group differences disappeared for all executive function variables (all *p* > 0.34, all η^2^*p* < 0.01; Supplementary Table 2) and most social cognition variables (all *p* > 0.14). The only difference that persisted was the recognition of sadness on the Mini-SEA [VC > NVC, *F*(1,75) = 6.420, *p* = 0.013, η^2^*p* = 0.08], consistent with the univariate finding. Crucially, incarceration length as a covariate was not significant in any model (all *p* > 0.39, all η^2^*p* < 0.01), confirming that it does not act as a confounding variable (see Table 5).

**TABLE 5:** ANCOVAs comparing VC and NVC groups controlling for incarceration length as a covariate.

Variable	Group effect (VC vs. NVC)	η ^2^p	Covariate effect (time)	η ^2^p(Cov)
WM sum (total)	*F*(1, 75) = 0.17, *p* = 0.683	0.002	*F*(1, 75) = 0.002, *p* = 0.962	< 0.001
Digits total	*F*(1, 75) = 0.90, *p* = 0.346	0.010	*F*(1, 75) = 0.04, *p* = 0.847	< 0.001
Number-letter	*F*(1, 75) = 0.02, *p* = 0.882	< 0.001	*F*(1, 75) = 0.01, *p* = 0.931	< 0.001
Disgust (mini-SEA)	*F*(1, 75) = 0.29, *p* = 0.593	0.004	*F*(1, 75) = 0.48, *p* = 0.491	0.006
Sadness (mini-SEA)	*F*(1, 75) = 6.42, *p* = 0.013*	0.080	*F*(1, 75) = 0.08, *p* = 0.777	0.001
Total score (mini-SEA)	*F*(1, 75) = 1.24, *p* = 0.268	0.020	*F*(1, 75) = 0.80, *p* = 0.375	0.010
Affective intentional	*F*(1, 74) = 2.12, *p* = 0.149	0.030	*F*(1, 74) = 0.66, *p* = 0.420	0.009
Empathetic concern (intent.)	*F*(1, 74) = 3.49, *p* = 0.066	0.040	*F*(1, 74) = 0.69, *p* = 0.410	0.009

* denotes statistical significance at *p* < 0.05.

Finally, three-group ANCOVAs (VC, NVC, and Control; with incarceration length set to 0 for controls) were conducted to verify if the observed deficits were driven by the duration of confinement or by group membership. The analysis revealed that the effect of incarceration length was completely negligible for both the Working Memory Sum [*F*(1,113) = 0.003, *p* = 0.959, η^2^*p* < 0.001] and Number-Letter sequencing (*p* = 0.922). In contrast, the main effect of Group remained robust and significant [*F*(2,113) = 8.499, *p* < 0.001], with a medium effect size (η^2^*p* = 0.13). *Post-hoc* comparisons confirmed that the Control Group significantly outperformed both the Violent (*p* = 0.003) and Non-Violent (*p* < 0.001) groups, while no significant differences were observed between the two offender groups (*p* = 0.901). These findings conclusively demonstrate that working memory deficits are a shared characteristic of the offender population relative to non-offenders and are not an artifact of incarceration duration.

To assess potential confounding effects of incarceration length, analyses were restricted to the prisoner population, as the Control Group lacked this variable. Spearman correlations revealed no significant relationship between incarceration duration and any of the assessed cognitive variables (all | ρ| < 0.20, *p* > 0.11; see Supplementary Table 1). Notably, the Mini-SEA Total score showed a negligible correlation (ρ = -0.002, *p* = 0.984), indicating a complete absence of a linear association. These findings effectively rule out the possibility that the substantial difference in incarceration length between VC (*M* = 107 months) and NVC (*M* = 57 months) offenders accounts for the observed neurocognitive profiles.

To verify the robustness of the effects, multivariate analyses were conducted. Regarding executive functions, a one-way MANOVA on the WAIS-IV Digit Span subtests revealed a significant multivariate main effect of Group [Wilks’ Λ = 0.836, *F*(8, 222) = 2.61, *p* = 0.010], yielding a medium effect size (η^2^*p* = 0.08). Regarding social cognition, Permutational Multivariate Analyses of Variance (PERMANOVAs) on the Pain Task identified significant effects across all empathy-related thematic blocks. Specifically, significant differences were found in Empathetic Concern (*F* = 4.33, *R*^2^ = 0.07, *p* = 0.006), Cognitive Empathy (*F* = 4.16, *R*^2^ = 0.07, *p* = 0.002), Affective Empathy (*F* = 3.98, *R*^2^ = 0.07, *p* = 0.006), Intentionality Attribution (*F* = 3.65, *R*^2^ = 0.06, *p* = 0.008), and Personal Distress (*F* = 3.17, *R*^2^ = 0.05, *p* = 0.018). This specific pattern confirms that group differences are driven by deficits in empathetic processing rather than general moral judgment mechanisms.

## Discussion

This study reveals the complexity of the executive and cognitive functioning of the Colombian inmate population, showing that the differences between those who committed violent crimes (VC) and non-violent crimes (NVC) do not fully conform to the classic assumptions of the international literature. In describing the neuropsychological profiles of executive functions (EF), the findings indicate that, at a descriptive level, prisoners convicted of non-violent crimes tended to show lower scores than those convicted of violent crimes in tasks involving cognitive flexibility, although these differences did not reach statistical significance. In contrast, working memory showed significant group differences, with both offender groups performing below the CG. These patterns suggest that, while tendencies toward greater perseveration and difficulty in establishing categories were observed in the NVC group, the most robust executive deficit shared by both groups lies in the capacity to record, encode, and manipulate information. Similarly, in working memory (WM), there was evidence of difficulty in recording, encoding, and manipulating information, which affects the ability to maintain attention in the face of distractors. However, no significant differences were observed between groups in inhibitory control as assessed by the Stroop test, suggesting that the attentional difficulties may be more closely linked to working memory limitations than to a primary deficit in inhibitory capacity.

These results differ from most of the international research, which indicates a more impaired executive profile in VC, where the severity of the crime is associated with greater deficits in inhibitory control and self-regulation (Alvarado-Grijalba et al., 2020; Barker et al., 2007; Cruz et al., 2020; Fontao and Ross, 2021; Greenfield and Valliant, 2007; Leutgeb et al., 2016; Morgan and Lilienfeld, 2000; Ogilvie et al., 2011; Ross and Hoaken, 2011; Wallinius et al., 2019). Likewise, the literature has documented that violent inmates often exhibit significant alterations in behavioral inhibition and impulse control (Chaguendo-Quintero et al., 2023; Chen et al., 2014; Karlsson et al., 2016; Meijers et al., 2017; Slotboom et al., 2017; Vilà-Balló et al., 2014; Zou et al., 2013). The discrepancy found suggests that the classification between VC and NVC is not sufficient to explain executive performance, as this also appears to be conditioned by contextual, social, and personal variables (Aguilar-Cárceles, 2012; Baez et al., 2019).

Therefore, the results show that the incarcerated population is a heterogeneous group, with cognitive variations that may depend on educational level, previous occupation, the development of frontal circuits, and opportunities for cognitive stimulation throughout life (Álvis et al., 2014; Arana-Medina et al., 2019). These findings challenge the linear interpretation of the relationship between executive functions and type of crime, suggesting instead a multidimensional model that integrates neuropsychological factors with structural and social determinants. As Herrero et al. (2010) have also noted, not all criminal behavior stems from executive dysfunction, and therefore interventions must go beyond the strictly clinical sphere and take into account conditions of vulnerability, marginalization, and social exclusion.

When comparing the groups, WM was found to be significantly lower in the two groups of prisoners than in the control group (CG), but with similar performance between VC and NVC prisoners. This pattern is consistent with the findings of Hoaken et al. (2007), who also found reduced WM in prisoners with no differences between crime types. Carreño et al. (2018) suggest that this function is closely linked to violent behavior, given that the regulation of the stress response depends on the balance between the sympathetic and parasympathetic nervous systems. A dysfunction in the vagal pathway, which is responsible for modulating physiological responses to threats, can increase impulsivity and aggression, as it hinders the ability to process information and control emotions during stressful situations. From this perspective, working memory acts not only as a cognitive resource, but also as a mediator between emotional regulation and violent behavior.

These findings have important practical implications. Intervention programs targeting the prison population should include strengthening working memory and inhibitory control as components for preventing recidivism, considering that poor emotional self-regulation is a widely documented risk factor (Ligthart et al., 2019). Consequently, rehabilitation strategies should integrate stress regulation exercises, cognitive training, and emotional education, which promote both inhibition capacity and reflective processing of actions.

A further relevant aspect resides in the fact that the apparent absence of differences between VC and NVC in WM could reflect a functional homogenization resulting from the prison context. Prison routine, overcrowding, and lack of sustained cognitive stimulation have been described as potential factors contributing to executive decline in incarcerated populations. From an ecological perspective, the prison environment may act as a modulating factor capable of equalizing performance opportunities, potentially at a lower functional level.

Thus, prison neuropsychology should not only describe individual deficits, but also analyze how the institutional environment contributes to their maintenance or exacerbation. However, the present findings do not fully support the cognitive deterioration hypothesis associated with prolonged incarceration. Contrary to the cognitive deterioration hypothesis reported in European literature (Meijers et al., 2017, 2018), incarceration length neither correlated with cognitive performance nor acted as a significant covariate). This suggests that the observed deficits in working memory and empathetic processing reflect premorbid neurocognitive vulnerabilities rather than institutional decay. Four factors may explain this discrepancy: (1) the sample’s relative youth may offer greater cognitive reserve; (2) strict exclusion of severe psychopathology and current substance use removed vulnerable cases; (3) the incarceration duration may fall below thresholds for severe deterioration seen in extremely long-term confinement; and (4) the Colombian context of chronic structural violence may generate neurocognitive profiles distinct from those in low-violence regions. Theoretically, these findings indicate that cognitive differences are attributable to the offender profile rather than incarceration artifacts. This supports the view that executive deficits and heightened emotional resonance constitute premorbid risk markers for criminal behavior, rather than consequences of imprisonment.

These findings also suggest that we should rethink the traditional punitive approach and focus more on cognitive and social rehabilitation. If the differences between violent and nonviolent offenders do not appear to be so clear-cut in functional terms, the prison system should focus on identifying specific neuropsychological needs rather than classifications based on the legal nature of the crime. This finding reinforces the need for a multidimensional approach that combines neuropsychology with social psychology and criminology, integrating both cognitive processes and the contexts that shape them.

On the other hand, the apparent advantage of VC over NVC in tasks involving cognitive flexibility and inhibitory control could be interpreted as a functional adaptation rather than an indicator of cognitive superiority. Some studies have suggested that recurrent violent behavior could involve a form of “instrumental learning” from a hostile environment, in which the anticipation of threats or the need to respond immediately generates a type of involuntary training of certain executive functions. However, this apparent strengthening may coexist with deficits in moral regulation or affective integration of information, which would explain why these skills do not necessarily translate into prosocial behaviors.

Overall, the results show that EF in the prison population does not follow a simple pattern of deterioration proportional to the violence of the crime. Rather, it responds to a complex interaction between neurocognitive vulnerabilities and contextual adaptations, which must be understood using models that integrate biology, life history, and the structural conditions of the environment. In this regard, the differences found between the groups depart from the violent/nonviolent dichotomy paradigm, underscoring the need for approaches that are more sensitive to the Colombian sociocultural context, marked by a long history of structural violence (Cremades, 2021; González and Molinares, 2010).

In relation to social cognition (SC), the results further deepen the complexity of this phenomenon in the prison population. In the recognition of facial emotions, which is an essential skill for understanding and regulating human interactions (Bertoux et al., 2012; Švegar et al., 2019), a distinct pattern was observed depending on the type of emotion. Prisoners convicted of nonviolent crimes showed better performance in identifying negative emotions compared to the control group, while violent prisoners also performed above the control group average for these same expressions but slightly below for positive emotions. This finding is consistent with Zeng et al. (2022), who reported that prisoners convicted of violent crimes showed particular sensitivity to fear, suggesting that prolonged exposure to threatening contexts may have strengthened their ability to detect signs of danger.

However, the relationship between violence and emotional recognition cannot be interpreted in a linear fashion. In contrast to Chapman et al. (2018) and Seidel et al. (2013), who reported lower overall recognition in VC, the current results point to adaptive modulation: VC may have developed greater accuracy in identifying unpleasant emotions such as fear or sadness in contexts where such signals are key to social survival. This perceptual specialization could be a marker of socio-emotional adaptation, reflecting how previous experiences of violence shape the way in which others’ emotions are processed. In other words, a violent environment not only alters behavioral structures, but also shapes emotional sensitivity, directing attention toward signs of threat and reducing the empathic response to positive stimuli.

This pattern invites us to think of SC not as a fixed capacity, but as a flexible process that adjusts to the demands of the environment. The differences observed between VC and NVC could be interpreted as divergent adaptive trajectories, where differential exposure to hostile contexts generates particular forms of emotional reading. In this sense, the results broaden our understanding of the link between violence and social cognition, suggesting that emotional skills in criminal contexts do not always reflect impairment, but rather functional reorganizations that respond to environmental conditions.

When analyzing empathy, it was found that both VC and NVC exhibited high levels of emotional contagion in accidental and intentional situations, indicating an automatic affective response to the suffering of others. However, in cognitive empathy, the VC group performed better, which is contrary to what the literature has reported (Heynen et al., 2016; Mariano et al., 2017; Morrow, 2020; van Langen et al., 2014). This finding can be interpreted in two ways: on the one hand, it could reflect a sophisticated ability to understand the mental state of others a preserved or even developed cognitive ability; on the other hand, it could suggest the instrumental use of this ability, linked to social manipulation strategies. As Decety and Sveltova (2012) point out, cognitive empathy requires consciously putting oneself in another’s place, but does not necessarily imply prosocial motivation.

This interpretation becomes even more relevant when we observe that VC individuals showed low empathic concern in neutral situations, suggesting that their ability to understand the mental states of others does not translate into a genuine willingness to act in favor of others. This dissociation between understanding and motivation could reflect a functional disconnect between the prefrontal systems and limbic networks involved in affective regulation. Thus, certain violent individuals may understand the emotions of others but lack the motivational drive necessary to respond empathetically in everyday contexts. This mixed profile highlights the importance of including not only empathic abilities in prison assessments but also their motivational component, a fundamental aspect for guiding resocialization and recidivism reduction programs.

The findings regarding empathy once again challenge the dichotomy between violent and nonviolent individuals. The three groups (VC, NVC, and CG) showed contextual rather than structural differences. While the control group more easily recognized neutral situations, prisoners responded better to intentional or accidental scenarios, those charged with emotional or moral implications. This suggests that empathy in prisoners can be activated contingently in highly emotionally charged contexts, highlighting the plasticity of social and emotional processing and the possibility of stimulating it through contextualized interventions.

Furthermore, this study provides evidence from a country with unique sociopolitical and cultural conditions. In the case of Colombia, prolonged experiences of structural violence and inequality have shaped an emotional culture where exposure to pain and aggression is more frequent (Cremades, 2021; González and Molinares, 2010). Consequently, the normalization of violence could modulate the way in which the brain networks involved in social cognition process emotions and social interactions. Colombian prisoners, both violent and nonviolent, do not develop in neutral contexts, but in environments where the suffering of others and aggression are shared and persistent experiences. This could explain the apparent homogeneity of their empathic and cognitive profiles, as both groups are shaped by a cultural background of everyday violence.

Thus, neuropsychological evidence takes on a sociological and ethical perspective. The cognitive differences observed in prison populations reflect not only individual variations, but also the cumulative effects of inequality, exclusion, and institutional instability. As Baez et al. (2019) point out, violence cannot be understood outside the social context that produces and legitimizes it. In Colombia, factors such as poverty, limited access to opportunities, early exposure to violence, family neglect, and drug use (Aguilar-Cárceles, 2012; Denson, 2021; Anser et al., 2020) create an ecosystem conducive to criminal behavior. In this context, EF and SC are not simply individual traits, but expressions of a brain shaped by adversity and structural inequality.

From a methodological standpoint, this study strengthens its internal validity by matching groups according to age, education, and IQ, which allows for better isolation of the effects of the criminal context. However, limitations such as sample size and the cross-sectional nature of the study are acknowledged. Future work should incorporate longitudinal designs and the assessment of psychopathic traits or personality disorders to understand how they interact with EF and SC in the genesis and maintenance of violence (Cando-Pilatuña et al., 2019; Chaguendo-Quintero et al., 2023).

To summarize, the findings of this study challenge the rigidity of the violent/nonviolent classification and raise the need for an interpretive framework that articulates the neuropsychological with the sociocultural variables. Suggesting that cognitive and socio-emotional functioning may be more relevant indicators of behavioral risk and rehabilitation potential than offense categories alone. In doing so, this research advances an emerging international perspective that views crime not merely as a legal violation but as the product of dynamic interactions between brain function, developmental trajectories, and chronic exposure to social adversity (violence, inequality, and lack of opportunities). Recognizing this complexity requires rethinking the prison system and its intervention models, orienting them toward a more humanized and scientific view of crime.

Furthermore, by highlighting the cognitive consequences of punitive and overcrowded prisons, this study contributes to global debates on incarceration, advocating for policies that align with international standards on mental health, human rights, and violence prevention. Understanding the relationship between executive functions, social cognition, and the environment allows for the design of more comprehensive rehabilitation programs. These should include cognitive stimulation, emotional education, and social reintegration from a restorative justice approach. Such an approach recognizes that crime does not arise in a vacuum, but rather from the convergence of biological vulnerabilities and structural inequalities.

Finally, it is necessary to critically examine the tendency to understand of criminal behavior to dichotomous categories, as this impoverishes the debate and hinders the creation of effective solutions. In the Colombian context, where violence is historical and structural, neuropsychological findings take on an ethical connotation. Challenging the violent/nonviolent classification implies questioning a system that labels, segregates, and reproduces the very conditions that give rise to crime. Therefore, studying executive functions and social cognition not only contributes to science but also opens up the possibility of rethinking prison policies from the perspective of human dignity, prevention, and social equity.

## Data Availability

The datasets presented in this study can be found in online repositories. The names of the repository/repositories and accession number(s) can be found below: The data supporting this study are publicly available in the institutional repository of the Universitat Autònoma de Barcelona (UAB), as part of the author’s doctoral thesis. The dataset can be accessed through the UAB repository at http://hdl.handle.net/10803/693056.
