# Supplementary material for: Rewriting the crime divide: how executive functions and social cognition processes challenge violence classifications in the Colombian prison population
Source: Front Psychol. 2026 Apr 1;17:1741171. doi: 10.3389/fpsyg.2026.1741171 (PMC13080389; doi:10.3389/fpsyg.2026.1741171)
Supplement: Supplementary file 1 [file Table_1.docx]

Supplementary Material

# Supplementary Data

Supplementary Material should be uploaded separately on submission. Please include any supplementary data, figures and/or tables.

Supplementary material is not typeset so please ensure that all information is clearly presented, the appropriate caption is included in the file and not in the manuscript, and that the style conforms to the rest of the article.

# Supplementary Figures and Tables

For more information on Supplementary Material and for details on the different file types accepted, please see [here](https://www.frontiersin.org/guidelines/author-guidelines#supplementary-material).

## Supplementary Figures

**Figure 1**

*Profile of EFs in prisoner groups*

*Note.* Violent Crimes (VC); Non-Violent Crimes (NVC); Correct categories (WCST-C); Perseverations (WCST-P); Errors (WCST-E); Digits (WAIS IV-D); Numbers and Letters (WAIS IV-NL); Working Memory Sum (WAIS IV-WM); Word (STROOP-W); Color (STROOP-C); Word-Color (STROOP-WC); Interference (STROOP-I).

**Figure 2**

*Profile of SC in prisoner groups*

*Note.* Violent Crimes (VC); Non-Violent Crimes (NVC); Total score (Mini-SEA PT); Negative emotions (Mini-SEA EN); Positive emotions (Mini-SEA EP); Neutral (N); Accidental (A); Intentional (I).

## Supplementary Tables

**Table 1**

*Sociodemographic and screening variables*

| **Variables** | **DV** | | **DNV** | | **GC** | |
| --- | --- | --- | --- | --- | --- | --- |
|  | *M* | *SD* | *M* | *SD* | *M* | *SD* |
| Age | 32.95 | 5.7 | 30.23 | 6.45 | 29.05 | 8.92 |
| Years of education | 10.94 | 1.19 | 10.76 | 1.45 | 11.69 | 1.84 |
| Number of times in prison | 1.18 | 0.51 | 1.46 | 0.85 | - | - |
| Time spent in prison in months *** | 69.23 | 31.01 | 38.01 | 28.37 | - | - |
| Anxiety (BAI) | 3.08 | 3.77 | 2.95 | 4.96 | 3.77 | 4.84 |
| Depression (BDI) | 4.64 | 6.17 | 4.1 | 4.42 | 5.85 | 5.37 |
| Intelligence Quotient (TAP) | 17.77 | 4.18 | 15.72 | 3.87 | 17.21 | 4.2 |

****p* < 0.001. Mean (M); Standard Deviation (SD); Violent Crimes (VC); Non-Violent Crimes (NVC); Control Group (CG).

**Table 2**

*Comparison between groups in EF tests*

| Executive functions | Subvariables | VC | | NVC | | CC | | H/f | P-value |
| --- | --- | --- | --- | --- | --- | --- | --- | --- | --- |
|  |  | *M* | *SD* | *M* | *SD* | *M* | *SD* |  |  |
| Flexibility  (M-WCST) | Correct categories | 5.08 | 1.29 | 4.79 | 1.51 | 5.15 | 1.23 | 1.27 | 0.52 |
|  | Perseverations | 4.85 | 4.25 | 5.15 | 5.09 | 3.56 | 3.14 | 1.58 | 0.45 |
|  | Errors | 10.15 | 7.9 | 11.69 | 8.4 | 8.64 | 6.26 | 2.32 | 0.31 |
| Working memory (WM-WAIS-IV) | Digits | 24.38 | 3.05 | 23.39 | 3.35 | 26.1 | 3.21 | 5.86 | 0.00*** |
|  | Number-letters | 15.56 | 3.35 | 15.69 | 4.14 | 18.18 | 2.32 | 16.33 | 0.00*** |
|  | WM sum | 39.95 | 5.8 | 39.38 | 6.26 | 44.28 | 5.01 | 15.85 | 0.00*** |
| Inhibitory control (STROOP) | Word (W) | 98.23 | 16.01 | 95.54 | 16.34 | 99.77 | 14.51 | 0.73 | 0.48 |
|  | Color (C) | 66.03 | 9.55 | 66.74 | 10.41 | 66.95 | 12.95 | 0.07 | 0.92 |
|  | Word-color (WC) | 39 | 10.26 | 38.26 | 8.97 | 40.72 | 9.41 | 0.67 | 0.50 |
|  | Interference (I) | 0.94 | 6.93 | -0.81 | 7.3 | 9.31 | 2.45 | 0.58 | 0.55 |

***p < 0.001. Mean (M); Standard deviation (SD); Violent Crimes (VC); Non-Violent Crimes (NVC); Control group (CG).

**Table 3**

*Comparison between groups in facial emotions recognition*

| Facial Emotions  (Mini-SEA) | VC | | NVC | | CG | | H | P-value |
| --- | --- | --- | --- | --- | --- | --- | --- | --- |
|  | *M* | *SD* | *M* | *SD* | *M* | *SD* |  |  |
| Fear | 1.82 | 1.35 | 2.26 | 1.23 | 1.69 | 1.38 | 3.73 | 0.15 |
| Disgust | 2.9 | 1.33 | 3.38 | 1.23 | 2.74 | 1.19 | 6.11 | 0.04* |
| Anger | 3 | 1.08 | 3.13 | 1 | 2.74 | 1.23 | 2.69 | 0.25 |
| Surprise | 4.05 | 1.28 | 4.41 | 0.85 | 4.38 | 1.14 | 2.32 | 0.31 |
| Sadness | 3.41 | 1.31 | 2.97 | 1.39 | 2.67 | 1.26 | 6.25 | 0.04* |
| Neutral | 4.49 | 1.07 | 4.51 | 0.91 | 4.54 | 0.79 | 0.41 | 0.81 |
| Joy | 4.92 | 0.27 | 4.79 | 0.41 | 4.85 | 0.43 | 2.61 | 0.26 |
| Total score | 24.59 | 3.96 | 25.46 | 3.93 | 23.62 | 3.75 | 7.38 | 0.02* |
| Negative emotions | 11.13 | 2.73 | 11.74 | 2.75 | 9.85 | 3.13 | 7.70 | 0.02* |
| Positive emotions | 8.97 | 1.35 | 9.21 | 0.98 | 9.23 | 1.39 | 1.93 | 0.38 |

*p < 0.05. Mean (M); Standard deviation (SD); Violent Crimes (VC); Non-Violent Crimes (NVC); Control group (CG).

**Table 4**

*Comparison between groups in empathy components*

| Components | Situations | VC | | NVC | | CG | | f | P-value |
| --- | --- | --- | --- | --- | --- | --- | --- | --- | --- |
|  |  | *M* | *SD* | *M* | *SD* | *M* | *SD* |  |  |
| Affective Empathy | Neutral | -6.23 | 2.87 | -4.27 | 2.87 | -5.10 | 3.63 | 2.56 | 0.18 |
|  | Intentional | 7.59 | 1.78 | 6.70 | 1.78 | 4.73 | 3.65 | 8.85 | 0.00*** |
|  | Accidental | 2.73 | 4.09 | 3.80 | 4.09 | 1.41 | 4.68 | 2.76 | 0.07 |
| Cognitive Empathy | Neutral | 32.29 | 16.01 | 33.82 | 16.01 | 43.01 | 8.19 | 7.18 | 0.00*** |
|  | Intentional | 51.28 | 3.09 | 49.62 | 3.09 | 47.97 | 7.89 | 2.74 | 0.10 |
|  | Accidental | 33.99 | 8.14 | 30.51 | 8.14 | 31.22 | 13.85 | 0.87 | 0.82 |
| Empathetic Concern (motivational) | Neutral | -6.26 | 2.87 | -4.45 | 2.87 | -5.00 | 3.73 | 2.99 | 0.20 |
|  | Intentional | 7.73 | 2.11 | 6.46 | 2.11 | 4.42 | 4.00 | 9.46 | 0.00*** |
|  | Accidental | 3.51 | 4.24 | 4.36 | 4.24 | 1.84 | 4.62 | 2.99 | 0.03* |

*p < 0.05, ***p < 0.001. Mean (M); Standard deviation (SD); Violent Crimes (VC); Non-Violent Crimes (NVC); Control group (CG).
